# Examples of overlooking common sense solutions: the domestication gene and selection against mortality

**DOI:** 10.3389/fgene.2014.00266

**Published:** 2014-08-07

**Authors:** Jeroen van Rooijen

**Affiliations:** Retired: Former Poultry Behavior Researcher Center for Applied Poultry Research, Het SpelderholtBeekbergen, Netherlands

**Keywords:** bankiva, Ceylon jungle fowl, domestication locus, mortality selection, feather pecking

Today the developments in genetics are exciting. Perhaps this explains why geneticists sometimes seem to overlook common sense solutions. One example of this is the selection experiment done by Bijma et al. (2007a,b). These authors developed a sophisticated statistical method of group selection against mortality in hens randomly placed together. One may safely assume that this mortality is due to cannibalism as a result of severe feather pecking. However, they seemed to overlook the possibility that severe feather pecking is no normal behavior performed by all individuals. As a result their selection seemed not very efficient. It is often assumed that layers selected for production traits show severe feather pecking because more aggressive hens have more opportunities to obtain food. Sufficient food is necessary for a good production. Therefore, aggressive hens are supposed to have a higher fitness during the selection for production traits resulting in an undesirable correlated selection response for this trait. However, aggression does not explain why in other strains selected for production traits severe feather pecking is almost absent. It does also not explain why in species not selected for production traits severe feather pecking occurs frequently under husbandry conditions. For instance, game pheasants, partridges, ostriches and in the wild ancestor of our domestic fowl: the bankiva (*Gallus gallus*; Van Rooijen, 2010a). Severe feather pecking differs from aggressive pecking in several respects, for instance, aggressive pecks are always aimed at the head, whereas severe feather pecks are aimed at other body parts (Savory, 1995). Unlike aggressive pecking, severe feather pecking is not performed by all individuals but it is a deviant behavior performed by particular individuals (Keeling, 1994). The aim of selection under commercial conditions must be to select against these individuals (Van Rooijen, 2010b).

Another example of overlooking a common sense solution is the experiment performed by Rubin et al. (2010). To reveal loci under selection during domestication, these authors resequenced the whole genome of groups of domestic poultry and that of bankiva jungle fowl. These authors found that essentially all individuals of domesticated fowl carry a TSHR allele (the locus for the thyroid stimulating hormone receptor). The TSHR-gene plays a role in the photoperiod control of reproduction, i.e. it explains why domestic hens lay eggs almost the year round. In bankiva zoo populations this allele was only found at intermediate frequency. Therefore, these authors concluded that TSHR may be a domestication locus in chicken. They assumed that the presence of this deviant TSHR-gene in bankiva zoo populations was due to hybridization with domestic chicken. Apparently they overlooked the possibility that this gene could originate from the Ceylon jungle fowl (*Gallus lafayetti*), otherwise they had incorporated this fowl in their study. There is a good reason to consider this possibility. During the breeding season, to attract females, the male bankiva is less camouflaged than the female. Outside the breeding season the plumage of the bankiva cock is more hen-like (eclipse plumage). The domestic cock does not possess such an eclipse plumage and is able to reproduce the year round. Probably, the TSHR-gene plays also a role in the photoperiod control of the male eclipse plumage, i.e., it probably explains why domestic males have a similar plumage the year round. Also the Ceylon male does not possess an eclipse plumage. The Ceylon male and female seem able to reproduce the year round (this is confirmed by fancy fowl keepers) and, thus, oviposition and male sexual behavior seem hardly controlled by photoperiod. The explanation may be that Ceylon fowl lives on Sri Lanka (formerly Ceylon), where from May to August the Yala monsoon brings rain to the south western half of the island, and from October to January the Maha monsoon to the North and East (Cummings et al., 2006). Therefore the relation between day length and the most suitable period for reproduction is not straightforward for the Ceylon jungle fowl population on Sri Lanka. This suggests that the Ceylon jungle fowl carry the deviant TSHR-locus. Probably, the domestic chicken has a multiple origin (Nishibori et al., 2005). For instance, the gene for the yellow skin descents from the Sonnerats jungle fowl (*Gallus sonneratii*) (Eriksson et al., 2008. Therefore one may question whether the TSHR-locus does not descend from Ceylon jungle fowl and thus is much older than domestication. The suggestion that the TSHR-locus is a domestication locus seems premature, due to a focus on the genetic method.

## Conflict of interest statement

The author declares that the research was conducted in the absence of any commercial or financial relationships that could be construed as a potential conflict of interest.
